# Leptospirosis infections among hospital patients, Sarawak, Malaysia

**DOI:** 10.1186/s40794-021-00154-2

**Published:** 2021-11-01

**Authors:** King-Ching Hii, Emily R. Robie, Izreena Saihidi, Antoinette Berita, Natalie A. Alarja, Leshan Xiu, James A. Merchant, Raquel A. Binder, Johnny Keh-Tun Goh, Vanina Guernier-Cambert, Diego Galán, Michael J. Gregory, Gregory C. Gray

**Affiliations:** 1 Department of Pediatrics, Kapit Hospital, Ministry of Health Malaysia, Kapit, Sarawak Malaysia; 2 Duke Global Health Institute, Duke University, Durham, NC USA; 3 Division of Infectious Diseases, Duke University School of Medicine, DUMC Box 102359, Durham, NC 27710 USA; 4 National Health Commission Key Laboratory of Systems Biology of Pathogens, Institute of Pathogen Biology, Chinese Academy of Medical Sciences and Peking Union Medical College, Beijing, China; 5 Department of Biostatistics and Bioinformatics, Duke University, Durham, NC USA; 6 Present address: Agricultural Research Service, National Animal Disease Center, United States Department of Agriculture, Ames, IA USA; 7 United States Naval Medical Research Center- Asia, Singapore, Singapore; 8 Emerging Infectious Disease Program, Duke-NUS Medical School, Singapore, Singapore; 9 Global Health Center, Duke Kunshan University, Kunshan, China

**Keywords:** Leptospirosis, Sarawak, Malaysia, Diagnostics

## Abstract

**Background:**

Leptospirosis diagnoses have increased in Sarawak, Malaysia in recent years.

**Methods:**

To better understand the burden of disease and associated risk factors, we evaluated 147 patients presenting with clinical leptospirosis to local hospitals in Sarawak, Malaysia for the presence of *Leptospira* and associated antibodies. Sera and urine specimens collected during the acute illness phase were assessed via a commercially available rapid diagnostic test (Leptorapide, Linnodee Ltd., Antrim, Northern Ireland), an ELISA IgM assay (*Leptospira* IgM ELISA, PanBio, Queensland, Australia) and a pan-*Leptospira* real-time PCR (qPCR) assay to estimate disease prevalence and diagnostic accuracy of each method. Microagglutination testing was performed on a subset of samples.

**Results:**

Overall, 45 out of 147 patients (30.6%) showed evidence of leptospires through qPCR in either one or both sera (20 patients) or urine (33 patients), and an additional ten (6.8%) were considered positive through serological testing, for an overall prevalence of 37.4% within the study population. However, each diagnostic method individually yielded disparate prevalence estimates: rapid test 42.2% for sera and 30.5% for urine, ELISA 15.0% for sera, qPCR 13.8% for sera and 23.4% for urine. Molecular characterization of a subset of positive samples by conventional PCR identified the bacterial species as *Leptospira interrogans* in 4 specimens. A multivariate risk factor analysis for the outcome of leptospirosis identified having completed primary school (OR = 2.5; 95 CI% 1.0–6.4) and weekly clothes-washing in local rivers (OR = 10.6; 95 CI% 1.4–214.8) with increased likelihood of leptospirosis when compared with those who had not.

**Conclusion:**

Overall, the data suggest a relatively high prevalence of leptospirosis in the study population. The low sensitivities of the rapid diagnostic test and ELISA assay against qPCR highlight a need for better screening tools.

**Supplementary Information:**

The online version contains supplementary material available at 10.1186/s40794-021-00154-2.

## Background

Leptospirosis has been on the rise in Malaysia in recent years [1, 2], where it has been designated a notifiable disease since 2010. The disease is biphasic in nature, with acute flu-like symptoms at the onset lasting for about a week, followed by antibody production and excretion of leptospires in the urine in the second week of illness. Mild cases may present with little to no symptoms, but severe infections can damage vital organs, trigger cytokine storms, and lead to death [3, 4]. In Malaysia, a peak of 7806 confirmed cases and 92 deaths was reported in 2014 [2], which suggests that leptospirosis is a significant public health threat locally [1, 2, 5], particularly considering that many cases may be under-reported or misdiagnosed [5, 6].

Leptospirosis is a zoonotic bacterial infection that causes widespread morbidity and disproportionately impacts people in regions with warm, humid climates in which the bacteria can easily proliferate [3, 6]. It is caused by a gram-negative spirochete of the genus *Leptospira*, which has 64 known genomic species and 300 identified serovars [7, 8]. Early identification and treatment of leptospirosis is challenging as symptoms of infection are non-specific and easily confused with other more common causes of acute febrile illness.

Given limited capacity for culture and molecular testing in many clinical settings, leptospirosis is commonly diagnosed by serology. Microscopic agglutination testing (MAT) is considered the reference test for leptospirosis serological diagnosis, but it has low sensitivity in the early stage of infection until measurable levels of antibodies develop in the blood [9]. Such delays in diagnosis may lead to missing the critical window for administering antibiotics. In addition, MAT requires a panel of live leptospires as well as highly-trained personnel, and only a few laboratories in Malaysia are able to offer this technique [5]. Alternatively, commercially available enzyme linked immunosorbent assays (ELISAs) can be used to detect antibodies in sera, with the added advantage that they can be standardized [10]. Studies have found some ELISA assays to be more sensitive than MAT at identifying IgM presence during early stages of leptospirosis, particularly when paired sera are not available [3, 10].

When molecular testing is available, evidence of leptospires can be seen in sera samples before antibody levels become noticeably elevated [3, 10]. The bacteria can also be detected in cerebrospinal fluid and urine specimens as the infection progresses [3, 4, 11]; some studies have identified *Leptospira* excreted in urine samples within as little as seven days of symptom onset [11, 12]. Polymerase chain reaction (PCR) assays have been successful in detecting leptospires in both sera and urine, however, the specialized equipment and training required for this method make it impractical in lower-resourced laboratory settings where such assets are often not available.

Serological rapid diagnostic tests (RDTs) have been developed as user-friendly alternatives to these more rigorous laboratory diagnostics and are now commonly used to screen for suspected leptospirosis [10]. This is standard practice in Malaysia, where RDT-positive samples are further sent to a central laboratory for confirmatory MAT [2, 5, 7, 13, 14].

In this study, we sought to assess the diagnostic accuracy of commercially available RDT and ELISA assays against a newly developed pan-*Leptospira* real-time PCR molecular reference test [15] while estimating the prevalence of leptospirosis in Sarawak, Malaysia. We additionally tested the feasibility of using urine samples to diagnose suspected leptospirosis patients, and characterized disease presentation among the study population.

## Methods

### Study subject enrollment

Written consent was obtained from a parent and/or legal guardian of all participants under the age of 18. Children aged 7–18 were additionally asked to assent to study participation. Between February and September 2019, licensed medical officers at three primary hospitals in Sarawak, Malaysia identified patients that matched the U.S. Center for Disease Control and Prevention (CDC) case definition for clinical leptospirosis infection. Patients were invited to enroll in this cross-sectional study if they presented with either fever (axillary temperature measurement of more than 37.5°C by digital thermometer) or known exposure to *Leptospira* contaminated water, accompanied by at least two commonly associated symptoms of leptospirosis: myalgia, headache, jaundice, conjunctivitis, or rash [16]. Hospitals were located in both urban (Sibu Hospital) and rural settings (Kapit Hospital and Sarikei Hospital). Kapit, Sibu and Sarikei are located in a triangular point with intercity distances of 66 km to 222 km. Altogether, these three divisions cover 51,544.7 km^2^ and account for 42.4% of the land area of Sarawak.

Once informed consent was obtained from the patient and/or their legal guardian, trained study staff collected a blood sample, and instructed the participant in mid-stream urine collection procedures. Participants were additionally asked to provide basic demographic, symptomatology, and exposure risk information through the completion of a brief questionnaire.

### Sample processing

In the clinical laboratory, blood samples were left to coagulate for up to 60 min at room temperature before centrifugation at 2000×*g* for 10 min. Serum was aliquoted into three 2.0 ml cryovial tubes, with one immediately used for RDT screening, as described below. This aliquot was later sent for confirmatory testing if the RDT was found to be positive. Of the remaining serum, one aliquot was used to run an ELISA, and the other for nucleic acid extraction and real-time PCR (qPCR) analysis.

Urine samples were transferred to new sterile tubes and centrifuged at 800×g for 10 min to form a visible pellet before remaining supernatant was removed; if a visible pellet did not form, an estimated 100 μL of the sample was retained in the base of the tube. The urine sample was resuspended to a final volume of 300 μL in phosphate-buffered saline solution (PBS). A portion of the resuspended urine sample was used for the RDT assay and the remaining volume was aliquoted into 2.0 ml cryovial tubes for nucleic acid extraction and qPCR analysis.

All specimens were stored at − 80 °C until remaining tests were performed.

### Rapid diagnostic test

Initial RDT screening was performed by a laboratory technician at the enrolling hospital using a commercial latex agglutination test currently used for leptospirosis screening in Sarawak, Malaysia (Leptorapide, Linnodee Ltd., Antrim, Northern Ireland). This test is designed to detect IgM antibodies for the rapid serological diagnosis of human leptospirosis [17]. This screening was done separately using sera and urine specimens. Even though this RDT was designed for use with serum, both serum and urine samples were tested using the same procedure as per manufacturer instructions. Briefly, 5 μl each of the Leptorapide reagent and specimen were applied to the provided agglutination card and then mixed. The agglutination card was rotated gently for 2–3 min and the positive/negative result was interpreted by visual comparison with the included score card. Specimens with ambiguous results were run a second time, and considered inconclusive if still unclear.

### Microscopic agglutination test

In accordance with the Malaysian Ministry of Health protocol, acute sera samples with a positive RDT result were shared with the Institute for Medical Research in Kuala Lumpur, Malaysia for confirmatory testing. A positive MAT was defined as a single antibody titer ≥400, an equivocal MAT was defined as a single antibody titer between 1:50 and 1:200, and a negative MAT was defined as a single antibody titer < 1:50. In our study, convalescent sera samples were not available for MAT analysis.

### ELISA assay

Once acute sera samples were received, technicians performed a *Leptospira* IgM ELISA assay (PanBio, Queensland, Australia) per the manufacturer’s instructions. Briefly, patient sera and controls were diluted 1:100 in diluent reagent then added to *Leptospira* antigen coated microwells (serovar: Patoc). The plate was incubated for 30 min at 37 °C. After washing the plate with PBS, 100 μl of horseradish peroxidase-conjugated anti-human IgM was added and incubated further for 30 min at 37 °C. After a second PBS wash, 100 μl of the tetramethylbenzidine substrate was added and the plate was incubated for 10 min at room temperature. The reaction was stopped with 100 μl of 1 M phosphoric acid. The absorbance value of each well was read at 450 nm wave length and interpreted in terms of Pan-Bio units, calculated using the absorbance of positive control serum, negative control serum, and cut-off of calibrators provided by the manufacturer. A Pan-Bio unit ≥11 was considered positive for the presence of *Leptospira* IgM antibodies.

### qPCR

Total DNA was extracted from 140 μl each of the aliquoted sera and urine specimen according to the manufacturer’s instructions, using the QIAamp RNA Mini Kit (QIAGEN, Hilden, Germany) as recommended by El Bali, et al. [18]. The extracted DNA was then added to a master mix, containing 10 ul Sso Advanced Universal Probes Supremix (Bio-Rad, Hercules, California, USA), 4.4 µl ultra-pure water, and 0.2 µm each of 20 µM primers and probes targeting the 16S *Leptospira* gene, as described by Mohd Ali, et al. [15] in their pan-*Leptospira* qPCR assay, specifically designed for the Malaysian context. The amplification protocol consisted of 95 °C for 3 min followed by 50 cycles of 95 °C for 30 s and 61.3 °C for 30 s. As the qPCR results were not used for clinical diagnosis, a conservative approach was used to consider specimens with threshold levels (*Cq*) below 40.0 positive for infection. Positive and negative controls were included in each run.

### Molecular characterization

Extracted nucleic acid from specimen were shipped on dry ice to Duke University in North Carolina, USA for further characterization. At Duke, samples were screened by conventional PCR and, when positive (band at ~ 550 bp), Sanger sequencing (Eton Bioscience, Inc., Research Triangle, NC, USA) was done to determine the *Leptospira* genetic diversity at the specific level targeting the *secY* gene. Primers secY-F (5′-ATGCCGATCATTTTTGCTTC-3′) and secY-R (5′-CCGTCCCTTAATTTTAGACTTCTTC-3′) [19] were used to amplify 549-bp fragments. The PCR conditions consisted of a transcription step at 50 °C for 30 min and an initial denaturation step at 95 °C for 15 min, followed by 45 cycles of denaturation at 94 °C for 30 s, annealing between 50 and 58 °C for 30 s, and extension at 72 °C for 1 min, with a final extension at 72 °C for 10 mins, see standard operating procedure in supplemental information.

### Statistical analyses

Questionnaire data and laboratory results were entered into Microsoft Excel and then analyzed using SPSS Statistics version 21 (IBM, 2012) and R 3.5.1 (R Core Team, 2018), with *epitools* (Aragon, 2020), *epiR* (Stevenson, 2020)*, ggplot2* (Wickham, 2016) and *caret* (Kuhn, 2020) packages.

Participants were considered positive for leptospirosis if (i) either sera or urine showed evidence of *Leptospira* by qPCR, or (ii) at least two out of three serological tests (RDT, MAT, or ELISA) were positive. Sensitivity, specificity, and positive and negative predictive values (PPV, NPV) of the Leptorapide RDT and ELISA were calculated against both the combined and specimen-specific qPCR results. Correlation between tests was assessed using the phi coefficient.

Bivariate analysis using a student t-test and Fisher’s exact test were used to compare participant characteristics and their laboratory assays against the qPCR outcomes. Characteristics determined to be predictive of leptospirosis infection (*p*-value < 0.10) were incorporated into a generalized linear model, then further refined through stepwise, backward-elimination to identify statistically significant (p-value < 0.10) adjusted odds ratios.

## Results

### Participant characteristics

A total of 147 patients (88 male, 59 female) were enrolled across all three hospital sites (Table 1). Participant ages ranged from 2 to 78 years, with 56 participants (38.1%) under 12 years of age, 13 (8.8%) between 12 and 18 years, 71 (48.3%) between 18 and 65 years, and 7 (4.8%) above 65 years of age. Five different ethnicities were represented in the study, with the majority of participants at each location and in the overall study (122, 83.0%) identifying as Iban.

**Table 1:** Demographic and household characteristics of patients with febrile illness enrolled across three hospitals in Sarawak, Malaysia, between February and October 2019 (*n* = 147)

Characteristics	Kapit Hospital n (%)	Sarikei Hospital n (%)	Sibu Hospital n (%)	Total n (%)
Total	102 (69.4)	24 (16.3)	21 (14.3)	147 (100)
Gender				
Female	37 (36.3)	7 (29.2)	15 (71.4)	59 (40.1)
Male	65 (63.7)	17 (70.8)	6 (28.6)	88 (59.9)
Age				
Under 5	6 (5.9)	1 (4.2)	1 (4.8)	8 (5.4)
5–11	35 (34.3)	11 (45.8)	2 (9.5)	48 (32.7)
12–17	11 (10.8)	0 (0.0)	2 (9.5)	13 (8.8)
18–64	45 (44.1)	11 (45.8)	15 (71.4)	71 (48.3)
65 +	5 (4.9)	1 (4.2)	1 (4.8)	7 (4.8)
Education completed				
None	23 (21.6)	6 (25.0)	5 (23.8)	34 (23.1)
Primary	39 (38.2)	12 (50.0)	6 (28.6)	57 (38.8)
Secondary	36 (35.3)	5 (20.8)	8 (38.1)	49 (33.3)
College	2 (2.0)	0 (0.0)	2 (9.5)	4 (2.7)
Post-college	2 (2.0)	1 (4.2)	0 (0.0)	3 (2.0)
Ethnicity				
Iban	86 (84.3)	22 (91.7)	14 (66.7)	122 (83.0)
Chinese	2 (2.0)	1 (4.2)	2 (9.5)	5 (3.4)
Malay	4 (3.9)	1 (4.2)	1 (4.8)	6 (4.1)
Other	10 (9.8)	0 (0.0)	4 (19.0)	14 (9.5)
Type of housing				
House	37 (36.3)	5 (20.8)	7 (33.3)	49 (33.3)
Hut / Wood house	11 (10.8)	4 (16.7)	5 (23.8)	20 (13.6)
Longhouse	54 (52.9)	15 (62.5)	9 (42.9)	78 (53.1)
Primary drinking water source*				
River	55 (53.9)	8 (33.3)	2 (9.5)	65 (44.2)
Piped	44 (43.1)	14 (58.3)	14 (66.7)	72 (49.0)
Rain	10 (9.8)	4 (16.7)	3 (14.3)	17 (11.6)
Gravity feed	8 (7.8)	0 (0.0)	2 (9.5)	10 (6.8)
Other	1 (1.0)	0 (0.0)	1 (4.8)	2 (1.4)

* Some respondents gave multiple answers

Many participants lived in longhouses (78, 53.1%), with 49 (33.3%) reporting residency in houses, and 20 (13.6%) in wooden huts. Households frequently reported obtaining drinking water from multiple sources; 65 (44.2%) collected water from the river, 72 (49.0%) had water piped into their homes, 17 (11.6%) used rain water, and 10 (6.8%) had gravity feed water systems. River water was the most common drinking water source for participants enrolled at Kapit Hospital (55, 53.9%), while participants from Sarikei and Sibu Hospital more commonly reported using piped water (14 per site, 58.3 and 66.7%, respectively).

Among the study population, 34 (23.1%) had not completed any level of formal schooling, while 57 (38.8%) had completed primary and 49 (33.3%) completed secondary schooling. Only 7 (4.7%) participants had gone on to complete college or further professional studies. Among the 78 participants greater than 18 years of age, 46 (59.0%) were involved in occupations associated with high risk of leptospirosis infections, including agriculture (*n* = 29), fishing (*n* = 17), hunting (*n* = 11), and logging (*n* = 9). Many participants participated in multiple such activities. Within the study population, 52.6% of individuals greater than 18 years of age reported wearing closed-toe shoes for work (*n* = 41), while 30.8% wore open-toed shoes or sandals. Other reported personal protective equipment (PPE) usage included gloves (14.1%), eye protection (5.1%), and face masks (9.0%).

### Leptospirosis prevalence

**Fig. 1:**
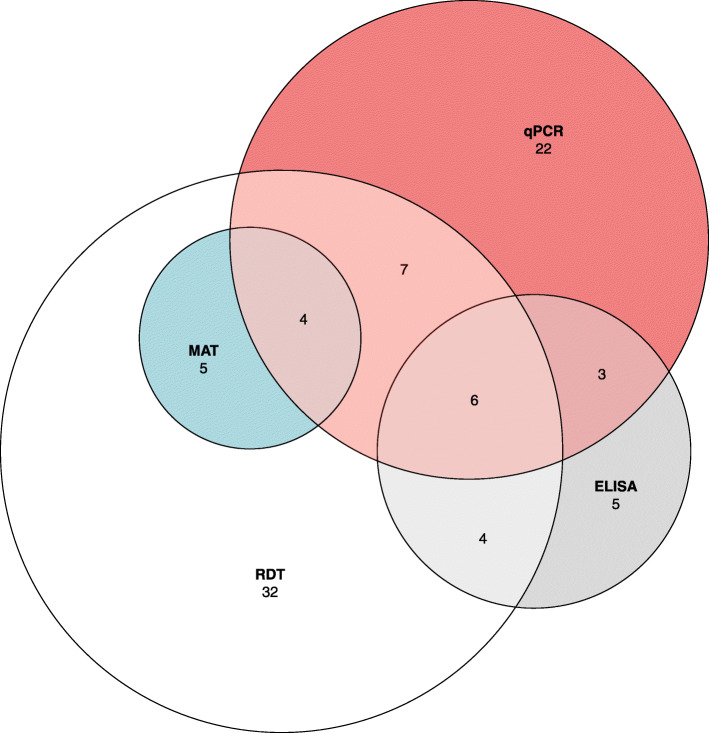
Comparison of positive serological leptospirosis results for patients presenting with febrile illness to study hospitals in Sarawak, Malaysia according to various diagnostic methods used. Participants were defined as having leptospirosis if at least two out of three serological tests were found to be positive and/or molecular evidence of *Leptospira* was identified through qPCR in either participant sera or urine. ^*^ RDT: Rapid diagnostic test, Leptorapide latex agglutination test (Linnodee, Ltd., Antrim, Northern Ireland). ^**†**^ MAT: Microagglutination testing, as carried out by the Institute of Medical Research reference lab in Kuala Lumpur, Malaysia, only partial results available. ^**‡**^ ELISA: *Leptospira* IgM ELISA assay (PanBio, Queensland, Australia)

Serological RDT and ELISA results were available for all 147 patients, with calculated prevalences of 42.2% and 15.0% respectively. Out of 62 positive RDT sera specimens, 53 were sent for confirmatory MAT results, along with 5 inconclusive RDT samples and 1 negative. Regardless of RDT outcome, 15/59 (25.4%) sera samples returned positive MAT results. Per our positivity criteria (at least two out of three serological tests positive), a total of 23 participants (15.6%) were considered serology-positive for leptospirosis, of which thirteen participants also presented molecular evidence of leptospires in either sera or urine (Fig. 1).

Two sera samples did not have sufficient volume to complete qPCR testing. Among 145 remaining sera and 141 matched urine specimens, a total of 45 leptospirosis infections were identified through qPCR (positive for either sera and/or urine). Evidence of *Leptospira* was found in 20 (13.8%) sera and 33 (23.4%) urine specimens.

Taken together, serological and molecular testing identified 55 patients with leptospirosis, resulting in an estimated prevalence of 37.4% (55 out of 147) within our study population (Table 2). At Kapit Hospital, 34 of 102 patients (33.3%) were considered positive for leptospirosis, as compared to 9 of 24 (37.5%) at Sarikei Hospital, and 12 of 21 (57.1%) at Sibu Hospital.

**Table 2:** Leptospirosis prevalence estimates among study population (n = 147) at three enrolling hospitals, Sarawak, Malaysia, according to various diagnostic tests employed. Participants were defined as having leptospirosis if at least two out of three serological tests were found to be positive and/or molecular evidence of *Leptospira* was identified in either participant sera or urine

Diagnostic type	Kapit Hospital n (%)	Sarikei Hospital n (%)	Sibu Hospital n (%)	Total n (%)
Sera				
Leptorapide RDT*	35/102 (34.3)	12/24 (50.0)	15/21 (71.4)	62/147 (42.2)
MAT^†^	2/31 (6.5)	6/14 (42.3)	7/14 (50.0)	15/59 (25.4)
ELISA assay^‡^	14/102 (13.7)	3/24 (12.5)	5/21 (23.8)	22/147 (15.0)
qPCR	16/102 (15.7)	3/23 (13.0)	1/21 (4.8)	20/145 (13.8)
Urine				
Leptorapide RDT*	32/100 (32.0)	4/22 (18.2)	7/19 (36.8)	43/141 (30.5)
qPCR	19/100 (19.0)	8/22 (36.4)	6/19 (31.6)	33/141 (23.4)
Leptospirosis-positive participants	34/102 (33.3)	9/24 (37.5)	12/21 (57.1)	55/147 (37.4)

^*^ Rapid diagnostic test: Leptorapide latex agglutination test (Linnodee, Ltd., Antrim, Northern Ireland). ^**†**^ Microagglutination testing, as carried out by the Institute for Medical Research reference lab in Kuala Lumpur, Malaysia, only partial results available. ^**‡**^ *Leptospira* IgM ELISA assay (PanBio, Queensland, Australia)

### Diagnostic accuracy

Leptorapide RDT analysis on patient sera samples identified 62 (42.2%) as positive, 67 (45.6%) as negative, and 18 (12.2%) inconclusive. RDT analysis on urine identified 43 specimens (30.5%) as positive and 98 (69.5%) as negative. Sensitivity, specificity, PPV, and NPV were assessed for each set of RDT analyses, using combined qPCR results for sera and urine as the gold standard (Table 3). MAT results were primarily available for RDT positive samples, and thus excluded from the diagnostic accuracy calculations to avoid bias; of note, 45.8% of MAT results were inconclusive, as compared to 25.4% positive and 28.8% negative findings *(Supplemental Fig.* *1**)*. Results of the Leptorapide RDT were further compared to specimen-specific qPCR results.

**Table 3:** Diagnostic accuracy of Leptorapide latex agglutination test (Linnodee, Ltd., Antrim, Northern Ireland) using acute sera and urine specimen, in comparison to real-time PCR (qPCR)

Diagnostic Type	Serum-based RDT*		Urine-based RDT*	
	RDT vs qPCR on sera or urine (95% CI†)	RDT vs qPCR on sera (95% CI†)	RDT vs qPCR on sera or urine (95% CI†)	RDT vs qPCR on urine (95% CI†)
Apparent prevalence	48.1 (39.2–57.0)	48.0 (39.1–57.1)	30.5 (23.0–38.8)	30.5 (23.0–38.8)
Sensitivity	47.5 (31.5–63.9)	36.8 (16.3–61.6)	22.2 (11.2–37.1)	18.2 (7.0–35.5)
Specificity	51.7 (40.8–62.4)	50.0 (40.2–59.8)	65.6 (55.2–75.0)	65.7 (56.0–74.6)
Positive predictive value	30.6 (19.6–43.7)	11.5 (4.7–22.2)	23.3 (11.8–38.6)	14.0 (5.3–27.9)
Negative predictive value	68.7 (56.2–79.4)	81.8 (70.4–90.2)	64.3 (54.0–73.7)	72.4 (62.5–81.0)

* Rapid diagnostic test. † Calculated confidence interval

The IgM ELISA assay identified 22 positive (15.0%), 113 negative (76.9%), and 12 equivocal specimens (8.2%). Sensitivity, specificity, PPV, and NPV of the IgM ELISA assay were calculated in comparison to combined qPCR results for sera and urine, as well as for sera-specific qPCR results (Table 4). As before, MAT results were excluded from this analysis.

**Table 4:** Diagnostic accuracy of *Leptospira* IgM ELISA assay (PanBio, Queensland, Australia) using acute sera specimen, in comparison to real-time PCR (qPCR)

Diagnostic Type	ELISA assay vs qPCR on sera or urine (95% CI)	ELISA assay vs qPCR on sera only (95% CI)
Apparent prevalence	16.3 (10.5–23.6)	16.4 (10.6–23.8)
Sensitivity	24.5 (13.3–38.9)	21.1 (6.1–45.6)
Specificity	88.4 (79.7–94.3)	84.3 (76.4–90.5)
Positive predictive value	54.5 (32.2–75.6)	18.2 (5.2–40.3)
Negative predictive value	67.3 (57.8–75.8)	86.6 (78.9–92.3)

### Molecular characterization

*SecY* sequencing was performed on samples showing PCR amplification and a clean band of the expected size (549 bp) on gel electrophoresis. Samples successfully sequenced (*n* = 4) originated from urine specimen collected within five days of fever onset. After cleaning and trimming, we compared the 410-bp fragments to *secY* sequences available from the MLST database (https://pubmlst.org/organisms/leptospira-spp) and identified *Leptospira interrogans* as the infecting species (GenBank accession numbers are provided in *Supplemental Table* *1**)*. When compared to a worldwide database including 134 *secY* sequences of *Leptospira interrogans* [20], our four sequences were identical or closely related to leptospires identified in Malaysia, Indonesia, India, the Philippines and China.

### Disease presentation

Among the 55 defined leptospirosis patients, 9.1% (*n* = 5) presented with severe disease, characterized by death (*n* = 1), septic shock (*n* = 4) and respiratory distress requiring invasive ventilation support (*n* = 3). Another seven (12.7%) presented with moderate disease requiring non-invasive ventilation support (n = 4), upper gastrointestinal bleeding (n = 1), atrial fibrillation (n = 1) and thrombocytopenia (*n* = 2). The remaining 43 patients (78.2%) presented with mild disease. Acute kidney injury and transaminitis were observed at all disease severity levels (34.5% and 32.7% respectively).

The most commonly self-reported symptoms among the leptospirosis-positive patients were fever (52, 94.5%), nausea or vomiting (39, 70.9%), headache (37, 67.3%), and chills (36, 65.5%) (*Supplemental Table* *2*). Several patients reported experiencing myalgia (29, 52.7%), joint pain (26, 47.3%), or conjunctivitis (17, 30.9%), while lethargy and diarrhea were uncommon (3, 5.5% and 9, 16.4%, respectively). Notably, jaundice was not observed in any of the leptospirosis positive patients.

Of note, two additional study participants died during the course of their hospitalization, but we found no detectable evidence of *Leptospira* infection in either of their samples.

### Symptom onset

**Fig. 2:**
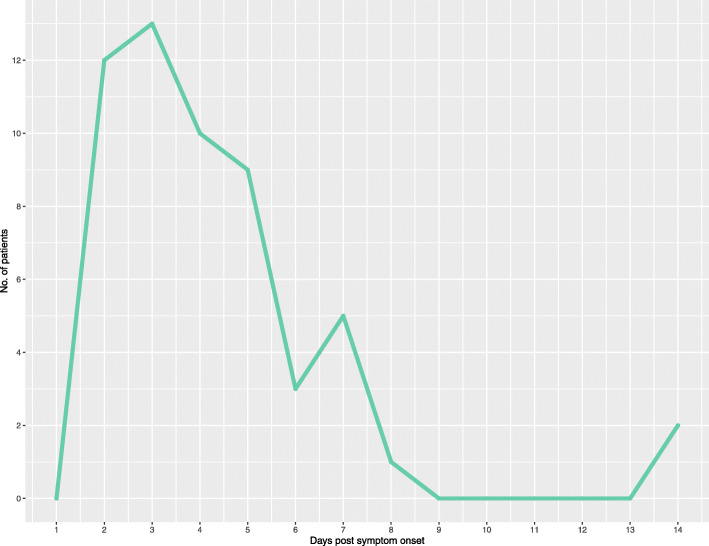
Days post symptom onset (DPSO) at the time of enrollment, as self-reported by leptospirosis patients (diagnosis determined by molecular and/or serological evidence). The acute phase typically takes place in the first week of illness, followed by antibody development 5–7 days post symptom onset

Leptospirosis-positive patients generally presented in the early phase of the disease, indicating that they were likely in the acute stage of infection at the time of sample collection (Fig. 2). Overall, 47 of 55 defined positive patients (85.4%) were enrolled within 6 days post symptom onset (DPSO; self-reported), with five (9.1%) presenting on day 7 and one (1.8%) on day 8. Two patients did report having experienced joint pain and myalgia, among other symptoms, for 14 days.

Comparatively, patients considered negative for leptospirosis reported having experienced non-specific febrile symptoms from one to 28 days prior to sample collection.

**Fig. 3:**
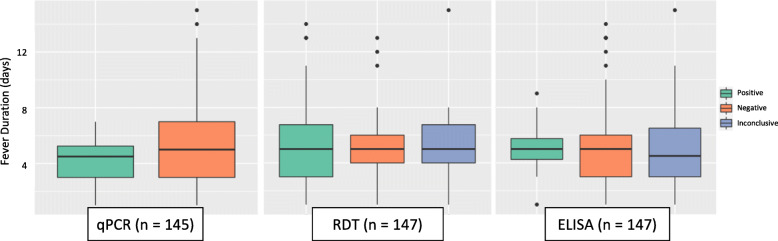
Evidence of leptospirosis in sera specimens from different diagnosis methods and according to patient-reported time of fever duration at time of sampling. Diagnosis determined by real-time PCR analysis, Leptorapide latex agglutination test (Linnodee, Ltd., Antrim, Northern Ireland), or *Leptospira* IgM ELISA assay (PanBio, Queensland, Australia)

**Fig. 4:**
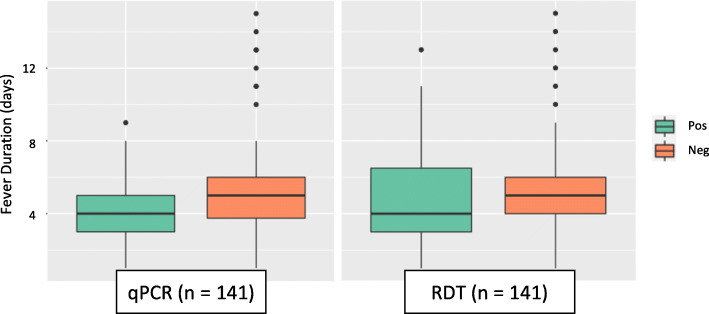
Evidence of leptospirosis in urine specimens from different diagnosis methods and according to patient-reported time of fever duration at time of sampling. Diagnosis determined by real-time PCR analysis or Leptorapide latex agglutination test (Linnodee, Ltd., Antrim, Northern Ireland)

Correlation calculations identified sera and urine specimen qPCR analysis as complementary (*Supplemental Table* *3*). Of the 45 participants with molecular evidence for leptospirosis, 12 (26.7%) were qPCR positive only through sera analysis, 25 (55.6%) only through urine, and 8 (17.8%) had *Leptospira* positivity in both sample types (Table 5*)*. Sera-based qPCR results primarily picked up evidence of *Leptospira* presence between 2 and 6 DPSO, with one patient testing positive with no documented fever (Fig. 3). Urine-based qPCR results were positive between 1 and 8 DPSO, with three patients testing positive with no documented fever (Fig. 4).

**Table 5:** Likely phase of disease progression for leptospirosis qPCR-positive patients as defined by sample positivity in sera and/or urine specimens. Participants with *Leptospira* evidence only in sera were classified to be in the onset phase, those with evidence in both sera and urine were classified in the dissemination phase, and those with evidence only in urine were classified in the excretion phase

Phase	No. of Patients	DPSO* Range	Sera Mean Cq^†^ (Range)	Urine Mean Cq^†^ (Range)
Onset	12	2–6	37.16 (35.36–39.45)	–
Dissemination	8	0–5	36.57 (34.80–39.60)	35.77 (27.17–39.85)
Excretion	25	0–8	–	37.38 (29.23–39.96)

*Days post-symptom onset (DPSO): fever duration at time of enrolment and sampling as self-reported by the patient. ^**†**^Real-time PCR-determined cycle threshold (Cq)

Participants with serological evidence for leptospirosis (*n* = 23) were identified from 1 to 8 DPSO, with one identified at 14 DPSO and one reporting no fever. Among these, the ten patients with no molecular evidence of *Leptospira* were primarily identified between 2 and 7 DPSO, with one patient presenting at 14 DPSO.

Independently of other serological assessments, the Leptorapide RDT provided positive results for patients 1 to 20 DPSO using sera samples and 1 to 14 DPSO using urine samples. The majority of the urine-based RDT positive results were within 1 to 7 DPSO, with 1 specimen positive at 10 DPSO and another at 14.

The IgM ELISA assay detected antibody evidence in patients 2 to 8 DPSO, and in one participant presenting without fever. MAT positives were identified primarily in participants 2 to 7 DPSO, as well as in one participant at day 10, one at day 14, and one presenting without fever.

### Risk factor analysis

Bivariate analyses demonstrated the participant’s completed level of education, type of housing, and frequency of washing clothes in the local river as being associated with leptospirosis diagnosis. Participant ethnicity, occupation, use of PPE, primary water source, and exposure to cats, dogs, pigs, poultry, or rodents were not found to be significantly associated with leptospirosis diagnosis, nor was the enrollment site, recent flooding, engaging in other river-based activities, household income, or primary mode of transportation (*Supplemental Table* *4*).

Multivariate analysis identified completing primary school and weekly washing of clothes in the local river as most predictive of a positive leptospirosis result in sera and/or urine. In this joint model, participants who had completed primary school were more than twice as likely to test positive for leptospirosis infection when compared to those with no education (OR 2.5, 95% CI 1.0–6.4). Those who completed secondary school were nominally less likely to show evidence of infection than those with no education (OR 0.9, 95% CI 0.4–2.5) (Table 6).

**Table 6:** Predictive factors for positive leptospirosis diagnosis (having either molecular or serological evidence of leptospirosis) in the study population (n = 147), as calculated through bivariate and multivariate analyses

Risk Factor	Positive cases (%)	Negative cases (%)	Unadjusted OR* (95% CI†)	Adjusted OR* (95% CI†)
Education completed				
None (*n* = 34)	10 (29.4)	24 (70.6)	Ref‡	Ref‡
Primary (*n* = 57)	28 (49.1)	29 (50.9)	2.28 (0.95–5.86)	2.47 (1.00–6.41)
Secondary / College (*n* = 56)	17 (30.4)	39 (69.6)	1.04 (0.41–2.74)	0.95 (0.36–2.53)
Type of Housing				
House (*n* = 49)	23 (46.9)	26 (53.1)	Ref‡	–
Hut / Wood house (*n* = 20)	9 (45.0)	11 (55.0)	0.93 (0.32–2.67)	–
Longhouse (*n* = 78)	23 (29.5)	55 (70.5)	0.48 (0.22–1.00)	–
Washing clothes in river				
Daily (*n* = 24)	7 (29.2)	17 (70.8)	0.70 (0.25–1.78)	0.86 (0.30–2.27)
Weekly (n = 5)	4 (80.0)	1 (20.0)	6.03 (0.80–168.44)	10.56 (1.44–214.82)
None (*n* = 118)	44 (37.3)	74 (62.7)	Ref‡	Ref‡

* Odds ratio. † Calculated confidence interval. ‡ Reference category

Washing clothes in the local rivers on a daily basis resulted in similar odds of having a leptospirosis diagnosis when compared to those who reported never washing clothes in the river (OR 0.9, 95% CI 0.3–2.3). In contrast, individuals who washed clothes weekly in the rivers had a tenfold increase in the likelihood of leptospirosis diagnosis, albeit with very low specificity (OR 10.6, 95% CI 1.4–214.8).

Multicollinearity across the model was not identified as a concern, with a mean variance inflation factor of 1.06 for all included variables.

## Discussion

Our study data document the poor performance of the Leptorapide RDT assay compared to qPCR diagnosis. The screening tool displayed low sensitivity and specificity for both sera and urine specimen; over half the true cases would have gone unidentified through using only the RDT screening followed by MAT confirmation, as commonly prescribed in Malaysia. Insufficient data was available to provide effective comparison between the RDT and validated MAT results; the study of paired acute and convalescent sera samples would provide more conclusive MAT results and allow for better assessment of the antibody detection accuracy of the Leptorapide RDT. Nevertheless, the present results are consistent with other studies carried out in Southeast Asia [21, 22] which have likewise shown that sensitivity and specificity of RDTs for *Leptospira* infections are often low when compared to PCR.

When using qPCR as the gold standard test, the serum-based ELISA assay produced higher specificity than the RDT, but much lower sensitivity. Results for the ELISA test may be improved by testing paired sera samples rather than only acute stage samples [23], however this is more labor intensive and delays diagnosis.

There is a great need for more accurate, yet simple to perform diagnostic tests for leptospirosis in Southeast Asia (and other resource-restricted settings). PCR can confirm diagnosis in the early phase of the disease before antibody titers are at detectable levels, but appropriate laboratory support is lacking in many areas [24]. MAT testing is similarly resource intensive and restricted to reference laboratories, and misses a critical window as antibodies are lacking during the acute phase of the disease [5].

Our estimated prevalence data suggest that 37.4% (55 out of 147) of the patients who present with clinical signs and symptoms consistent with leptospirosis receive a positive laboratory confirmation. We recognize that this estimate has a number of limitations and may or may not represent true prevalence. However, our results support our position that leptospirosis is a frequent cause of illness in Sarawak, as reported in previous studies conducted in the region. From 2011 to 2013, Lela Suut et al. likewise found a population-based leptospirosis seroprevalence of 37.4% among 508 subjects among rural communities in the Rejang Basin of Sarawak, using ELISA and MAT [25]. They found evidence of three main serogroups detected in all three divisions: 22.1% Djasiman, 13.2% Shermani, and 7.9% Pomona [25]. A study conducted in urban areas of Sarawak describing the diversity of *Leptospira* spp. in rats and the environment identified four pathogenic species: *L. interrogans, L. weilii, L. borgpetersenii* and *L. noguchii* [26], while another study focusing on various rodent species in urban, suburban, and rural Sarawak identified *L. interrogans* and *L. borgpetersenii* [27]. In the present study, we did not perform serological typing but were able to characterize a subset of positive samples as infected by *L. interrogans*, a pathogenic species previously identified in human leptospirosis in the region [1, 28] and commonly reported worldwide.

To our knowledge, this is the first clinical study on human leptospirosis in Sarawak, East Malaysia. Most of the leptospirosis patients were enrolled during the first six days of fever onset, suggesting they were in the acute stage of the disease. Study data were remarkable in that no patients with laboratory evidence of leptospirosis infection exhibited jaundice, a finding that is inconsistent with previous reports from West Malaysia that reported higher prevalences (22.5% [29] and 4.9% [30]) of jaundice among their patients. As icteric leptospirosis is associated with higher mortality rates [3, 4], it is unsurprising that low patient mortality was attributed to acute infections in our patients, despite having been reported in other case series [29, 30].

Our data (*Supplemental Fig.* 2) support other studies that have also been able to detect *Leptospira* in urine during the acute phase (first week) of infection [11, 12]. Our study identified more urine samples as qPCR positive for leptospirosis than sera, which was unexpected as our samples were typically collected within a week post symptom onset. During the early stage of infection, *Leptospira* are most commonly investigated and identified within the blood stream [3, 7]. Bacterial presence can later be seen in both sera and urine samples as the infection disseminates and *Leptospira* colonize the kidneys. Finally, leptospires are cleared from the bloodstream during the convalescent phase, while still present in urine. As described by Esteves, et al., different sample types may indeed prove more effective for identifying *Leptospira* infection at varying stages of disease [31], with the inclusion of urine permitting a wider window of detection [3]. By testing paired sera and urine specimens, we were able to identify 25 additional leptospirosis cases, which would have been missed had only sera been analyzed.

Risk factor data were interesting in that, while weekly washing of clothes in the river greatly increased disease risk when compared to those not using the river for such activities, doing so daily had minimal effect on this same risk. This finding was fairly imprecise, given the low number of observances of weekly clothes washing, however it may reflect longer exposure time in *Leptospira*-contaminated river water, if one assumes that once-weekly washing correlates with a larger amount of clothes to process. Despite taking place in the same body of water, other river-based activities such as swimming, bathing, fishing, or commuting were not significantly associated with disease risk within the study population, regardless of frequency.

Completing primary school appeared to greatly increase the risk of leptospirosis in comparison to those with either no formal education or with more advanced training. Although household income and known occupational risk factors for leptospirosis were not found to significantly impact leptospirosis outcomes in our study population, the increased risk associated with having completed primary school may serve as a proxy for other socioeconomic exposures not studied here. Though the Malaysian Ministry of Health has reported greater rates of leptospirosis within 25–60 year old individuals [2], age was surprisingly not found to increase disease risk in our bivariate analysis and thus not incorporated into our multivariate modeling.

**Fig. 5:**
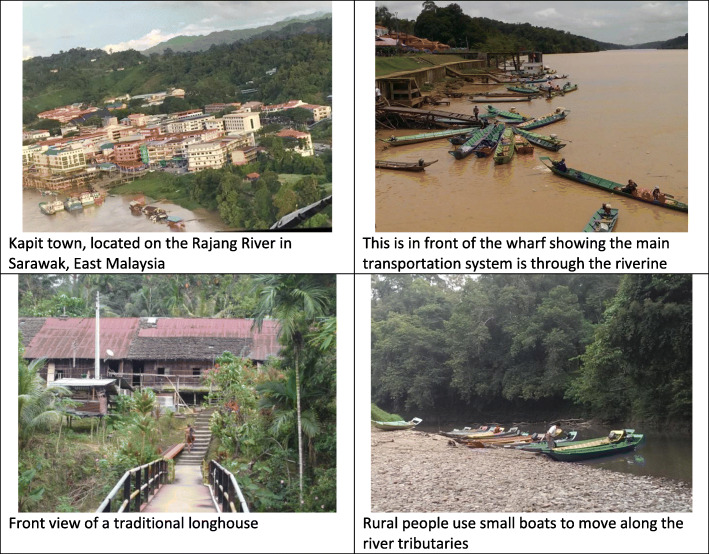
There are multiple potential sources of *Leptospira* infections in the region. Much of the area around Kapit Hospital has dense tropical forest and riverine systems rich in wildlife. This wildlife may introduce *Leptospira* into water systems. Most people live in longhouses or farm huts on the outskirts of the town of Kapit. Transportation is chiefly via boats on rivers. While Kapit is supplied with treated water, the source of the water in rural areas is either from river water or gravity fed rain-water-systems. Images here illustrate the living and transportation environments in which this study took place (photos provided by Dr. King-Ching Hii)

The study had a number of limitations. First it was a short-term study with limited geographical spread; we sampled for only a short time and only at three hospitals among the 22 hospitals in Sarawak so our findings may not be representative of the entire state of Sarawak. Further, in accordance with local clinical diagnostic procedures, MAT assays were only performed on a subset of the acute sera specimens that had positive RDT findings. Hence, our prevalence estimates may have underestimated true prevalence. Additionally, occupational exposure was difficult to assess, as only half the participants were of working age and few self-reported engaging with previously recognized high-risk occupations (e.g. fishing and agriculture). As people from rural Sarawak have many environmental water exposures which could serve as the source of *Leptospira* infection (Fig. 5), it is challenging to implicate specific risks and plans to study particular interventions. Finally, risk factor and symptom data were self-reported and could not be verified, offering the possibility of bias.

## Conclusion

This study confirms leptospirosis endemicity among the study population in Sarawak, Malaysia and identified low diagnostic performance of the commercial rapid diagnostic test and IgM ELISA assay studied when used during the acute phase of the disease. We conclude that there is a critical need for more sensitive, inexpensive, and easy to use rapid diagnostics for leptospirosis in this region. Until such diagnostics are available, clinicians in areas where molecular testing is unavailable will be wise to continue to treat empirically if the clinical suspicion of leptospirosis is high and RDTs are negative. Finally, our study found that in areas where PCR-based diagnostics are possible, testing both sera and urine can increase the probability of detecting leptospirosis during the acute phase.

## Supplementary Information

**Additional file 1.**

## Data Availability

The data collected during the current study are available from the corresponding author on reasonable request.

## Abbreviations

CDC: U.S. Center for Disease Control and Prevention
IgG: Immunoglobulin G
IgM: Immunoglobulin M
ELISA: Enzyme-linked immunosorbent assay
MAT: Microscopic agglutination testing
NPV: Negative predictive value
PBS: Phosphate-buffered saline solution
PCR: polymerase chain reaction
PPV: positive predictive value
RDTs: Serological rapid diagnostic tests
rPCR: real-time polymerase chain reaction
