# Supplementary material for: Leptospirosis infections among hospital patients, Sarawak, Malaysia
Source: Trop Dis Travel Med Vaccines. 2021 Nov 1;7:32. doi: 10.1186/s40794-021-00154-2 (PMC8559352; doi:10.1186/s40794-021-00154-2)
Supplement: Supplementary file 1 — Additional file 1. [file 40794_2021_154_MOESM1_ESM.docx]

**Supplemental Information**

Leptospirosis Infections Among Hospital Patients, Sarawak, Malaysia

King-Ching Hii,^1 ¶^ Emily R. Robie,^2,3 ¶^ Izreena Saihidi,^1^ Antoinette Berita,^1^ Natalie A. Alarja,^2,3^ Leshan Xiu,^2,3,4^ James A. Merchant,^5^ Raquel A. Binder,^2,3­­^ Johnny Keh-Tun Goh,^1^ Vanina Guernier-Cambert,^6^ Diego Galán,^3^ Michael J. Gregory,^7^ and Gregory C. Gray^2,3,8,9^ *

**Affiliations**

^1^ Department of Pediatrics, Kapit Hospital, Ministry of Health Malaysia, Kapit, Sarawak, Malaysia

^2^ Duke Global Health Institute, Duke University, Durham, North Carolina, USA

^3^ Division of Infectious Diseases, Duke University School of Medicine, Durham, North Carolina, USA

^4^ National Health Commission Key Laboratory of Systems Biology of Pathogens, Institute of Pathogen Biology, Chinese Academy of Medical Sciences and Peking Union Medical College, Beijing, China

^5^ Department of Biostatistics and Bioinformatics, Duke University, Durham, North Carolina, USA

^6^ Agricultural Research Service, National Animal Disease Center, United States Department of Agriculture, Ames, Iowa, USA

^7^ United States Naval Medical Research Center- Asia, Singapore

^8^ Emerging Infectious Disease Program, Duke-NUS Medical School, Singapore

^9^ Global Health Center, Duke Kunshan University, Kunshan, China

**S1:** **Enrollment Questionnaire**

Title of Study: **Leptospirosis and Associated Acute Febrile Illness Surveillance in Sarawak, Malaysia**

**Medical Facility (check one):** Hospital Sibu Hospital Kapit

**Today’s Date: ________________**(dd / mm / yy)

Date of Hospitalization: **________________**(dd / mm / yy)

The following to be completed by the patient, or in the case of a young child, by the parents/carers. Please check all that apply by marking an “X” in the box given:

**Patient’s Data and Demographics**

| - Male | - Female |
| --- | --- |

1. What is your (the child’s) gender?
2. What is your (the child’s) date of birth? **____________________** (dd / mm / yy)
3. What is your (the patient’s) ethnicity?

| - Iban | - Chinese | - Melanau | - Kenyah |
| --- | --- | --- | --- |
| - Malay | - Bidayuh | - Kayan | - Other: ___________ |

1. For the purposes of classifying your place of stay, what is your (the child’s) address/neighborhood

_____________________________________________________________________________________

1. What type of housing are you (child) living in?

|  | House | - Apartment | - Other: _______­­­­­_________ |  |  |
| --- | --- | --- | --- | --- | --- |
|  | Longhouse | - Hut |  |  |  |

1. Current house accommodations:

|  | Running water | - Hot water | - Concrete flooring |  |  |
| --- | --- | --- | --- | --- | --- |
|  | Stove top/oven | - Toilet | - Wood flooring |  |  |

1. What is your (the child’s) house primary source of water?

|  | Piped | - Gravity feed | - Other: _______­­­­­_________ |  |  |
| --- | --- | --- | --- | --- | --- |
|  | River | - Rain |  |  |  |

| - Yes | - No |
| --- | --- |

1. In the past month has your (the child’s) residency been affected by flooding?
2. Not including you (the child), how many individuals live in the patient’s household? ________
3. What is your (the child’s) highest level of education completed?

| - Primary | - College | - None |
| --- | --- | --- |
| - Secondary | - Post-College |  |

1. How many people in your (the child’s) household work (contribute to total household income)?

**Medical Data**

1. Are you pregnant (check one)?
   - No (or does not apply)
   - If yes, the expected due date is **____________________**(dd / mm / yy)
2. Are you (the child) experiencing any of the following symptoms? (If yes, list duration and indicate days/weeks)

|  |  |  |  |  |  |  |  |
| --- | --- | --- | --- | --- | --- | --- | --- |
|  |  | weeks | days |  |  | weeks | days |
|  | Fever |  |  |  | - Chills |  |  |
|  | Skin Rash |  |  |  | - Nausea/Vomiting |  |  |
|  | Jaundice |  |  |  | - Sore throat |  |  |
|  | Headache |  |  |  | - Conjunctivitis/red eyes |  |  |
|  | Joint Pain |  |  |  |  Other |  |  |
|  | Myalgia |  |  |  |  |  |  |

**Behavioral, Occupational and Environmental Risk Factors**

1. How much direct contact do you (the child) have with the Rajang river? Other river/body of water? ________________

| - Swimming | - Daily | - Weekly | - None |
| --- | --- | --- | --- |
| - Bathing | - Daily | - Weekly | - None |
| - Washing Clothes | - Daily | - Weekly | - None |
| - Fishing | - Daily | - Weekly | - None |
| - Other:__________________ | - Daily | - Weekly | - None |

1. In the last three (3) months, have you (the child) had physical contact with any of the following live animals?

|  | Pigs | - Poultry (chickens, ducks, geese, etc.) |
| --- | --- | --- |
|  | Cattle | - Cats |
|  | Sheep/goat | - Dogs |

| - Yes | - No |
| --- | --- |

1. In the last three (3) months, have you (the child) seen a rodent (rat, mouse, hamster etc.) where you live or work?

| - Yes | - No |
| --- | --- |

1. In the last three (3) months, have you (the child) handled a dead rodent (rat, mouse, hamster, etc.)?
2. What is your (the child) mode of transportation to work/school?

| - Express boat | - Long boat | - Motorbike | - Bicycle |
| --- | --- | --- | --- |
| - Car | - Van | - Foot | - Other: ___________ |

1. What is the type of route system used the most from your (the child) place of stay to place of activity (work/school)?

| - Tarred road | - Logging track | - Main river | - Other: |
| --- | --- | --- | --- |
| - Gravel road | - Small river | - Unpaved dirt road |  |

**For Adult Patients Over 18 Years of Age:**

1. What is your (patient) average monthly income?

| - None | - 600-1001 MYR | - 1501-2000 MYR | - Above 4000 MYR |
| --- | --- | --- | --- |
| - Below 600 MYR | - 1001-1500 MYR | - 2001-3000 MYR |  |

1. Out of the following options, which best describes the place in which you work?

| - - Mostly outdoors | - - Mostly indoors |
| --- | --- |
| - - A mix of outdoors and indoors | - - Unemployed |

1. Do you engage in any of the following for work?

|  | Logging industry | - Fishing |
| --- | --- | --- |
|  | Agricultural farming | - Hunting |
|  | Livestock farming | - Other job with exposure to animals: _______­­­­­_________ |

1. While working, do you wear any of the following?

|  | open-toe shoes (sandals) | - closed-toe shoes |
| --- | --- | --- |
|  | water-resistant boots | - latex or vinyl gloves |
|  | cloth or leather gloves | - glasses/protective eye wear |
|  | filtered facemask | - other personal protective equipment: _______________ |

Study Team Use:

Date of Discharge: **________________**(dd / mm / yy)

Results of Rapid Test in Site Hospital (Date: _____________): Positive / Negative

If MAT and/or PCR is done at Institute Medical Research (IMR) Centre, Kuala Lumpur, Malaysia.

Results of MAT (Date: _____________): Positive / Negative

Results of PCR (Date: _____________): Positive / Negative

**S1 Table: Sanger sequencing results for a subset of conventional PCR positive specimen targeting the *secY* gene.**[1] After cleaning and trimming, we compared the 410-bp fragments to secY sequences available from the MLST database (https://pubmlst.org/organisms/leptospira-spp).

| Sample | Medical Facility | Specimen type | Infecting *Leptospira* | GenBank Accession Number |
| --- | --- | --- | --- | --- |
| 1. | Kapit Hospital | Urine | *Leptospira interrogans* | MW228167 |
| 2. | Kapit Hospital | Urine | *Leptospira interrogans* | MW228168 |
| 3. | Sibu Hospital | Urine | *Leptospira interrogans* | MW228169 |
| 4. | Sarikei Hospital | Urine | *Leptospira interrogans* | MW228170 |

**S2 Table: Common symptoms associated with *Leptospira* infection, as experienced by patients with febrile illness determined to have molecular or serological evidence of leptospirosis.**

| Symptoms experienced | Positive cases  n = 55 (%) | Negative cases  n = 92 (%) | Total  n = 147 (%) |
| --- | --- | --- | --- |
| Fever | 52 (94.5) | 87 (94.6) | 139 (94.6) |
| Headache | 37 (67.3) | 66 (71.7) | 103 (70.1) |
| Chills | 36 (65.5) | 58 (63.0) | 94 (63.9) |
| Nausea / vomiting | 39 (70.9) | 52 (56.5) | 91 (61.9) |
| Myalgia | 29 (52.7) | 45 (48.9) | 74 (50.3) |
| Joint pain | 26 (47.3) | 34 (37.0) | 60 (40.8) |
| Conjunctivitis / red eyes | 17 (30.9) | 31 (33.7) | 48 (32.7) |
| Sore throat | 14 (25.5) | 20 (21.7) | 34 (23.1) |
| Diarrhea | 9 (16.4) | 10 (10.9) | 19 (12.9) |
| Loss of appetite | 9 (16.4) | 9 (9.8) | 18 (12.2) |
| Cough | 7 (12.7) | 8 (8.7) | 15 (10.2) |
| Abdominal pain | 7 (12.7) | 6 (6.5) | 13 (8.8) |
| Lethargy | 3 (5.5) | 10 (10.9) | 13 (8.8) |
| Reduced urine output | 3 (5.5) | 10 (10.9) | 13 (8.8) |
| Calf pain | 4 (7.3) | 4 (4.3) | 8 (5.4) |
| Dizziness | 2 (3.6) | 4 (4.3) | 6 (4.1) |
| Skin rash | 1 (1.8) | 4 (4.3) | 5 (3.4) |
| Jaundice | 0 (0.0) | 1 (1.1) | 1 (0.7) |
| Others | 3 (5.5) | 10 (10.9) | 13 (8.8) |

**S3 Table: Correlation of positive leptospirosis outcomes across various diagnostic tests employed on patients with febrile illness at three enrolling hospitals in Sarawak, Malaysia.** The qPCR urine and qPCR sera diagnostics complement each other as indicated by their negative correlation of -0.385. The urine RDT and Sera RDT tests have moderate positive correlation, indicating that both tests are correlated in terms of predicting positive cases.

|  | **Sera RDT** | **Urine RDT** | **Sera ELISA** | **qPCR Sera** | **qPCR Urine** | **MAT** |
| --- | --- | --- | --- | --- | --- | --- |
| **Sera RDT*** |  |  |  |  |  |  |
| **Urine RDT*** | -0.05 |  |  |  |  |  |
| **Sera ELISA^†^** | 0.32 | 0.04 |  |  |  |  |
| **qPCR Sera** | -0.27 | 0.07 | -0.18 |  |  |  |
| **qPCR Urine** | -0.12 | -0.10 | -0.07 | -0.39 |  |  |
| **MAT** | -0.01 | 0.18 | -0.18 | 0.10 | -0.03 |  |

**^*^** Rapid diagnostic test Leptorapide latex agglutination test (Linnodee, Ltd., Antrim, Northern Ireland). **^†^** Microagglutination testing, as carried out by the Institute of Medical Research reference lab in Kuala Lumpur, Malaysia, only partial results available. **^‡^** *Leptospira* IgM ELISA assay (PanBio, Queensland, Australia).

**S4 Table: Unadjusted odds ratios of *Leptospira* infection (as defined by molecular or serological evidence of leptospirosis) following exposure to common risk factors for leptospirosis.**

| Risk Factor | # Positive / # Total (%) | Unadjusted OR (95% CI) |
| --- | --- | --- |
| Medical facility | | |
| Kapit Hospital | 34 / 102 (33.3) | Ref |
| Sarikei Hospital | 9 / 24 (37.5) | 1.20 (0.46 – 3.02) |
| Sibu Hospital | 12 / 21 (57.1) | 2.63 (1.01 – 7.14) |
| Gender | | |
| Female | 25 / 59 (42.4) | Ref |
| Male | 30 / 88 (34.1) | 0.71 (0.36 – 1.40) |
| Age | | |
| Under 12 | 22 / 56 (39.3) | Ref |
| 12 – 17 | 7 / 13 (53.8) | 1.78 (0.51 – 6.38) |
| 18 – 64 | 23 / 71 (32.4) | 0.74 (0.35 – 1.55) |
| 65 + | 3 / 7 (42.9) | 1.17 (0.20 – 6.12) |
| Education completed | | |
| None | 10 / 34 (29.4) | Ref |
| Primary | 28 / 57 (49.1) | 2.28 (0.95 – 5.86) |
| Secondary / College | 17 / 56 (30.4) | 1.04 (0.41 – 2.74) |
| Ethnicity | | |
| Iban | 42 / 122 (34.4) | Ref |
| Chinese | 4 / 5 (80.0) | 6.82 (0.91 – 190.56) |
| Malay | 3 / 6 (50.0) | 1.89 (0.31 – 11.46) |
| Other | 6 / 14 (42.9) | 1.43 (0.44 – 4.48) |
| Type of housing | | |
| House | 23 / 49 (46.9) | Ref |
| Hut / Wood house | 9 / 20 (45.0) | 0.93 (0.32 – 2.67) |
| Longhouse | 23 / 78 (29.5) | 0.48 (0.22 – 1.00) |
| Primary water source* | | |
| River | | |
| Yes | 21 / 65 (32.3) | 0.68 (0.34 – 1.34) |
| No | 34 / 82 (41.5) | Ref |
| Piped | | |
| Yes | 29 / 72 (40.3) | 1.28 (0.63 – 2.62) |
| No | 26 / 75 (34.7) | Ref |
| Rain | | |
| Yes | 4 / 17 (23.5) | 0.49 (0.13 – 1.50) |
| No | 51 / 130 (39.2) | Ref |
| Gravity feed | | |
| Yes | 5 / 10 (50.0) | 1.73 (0.45 – 6.74) |
| No | 50 / 137 (36.5) | Ref |
| Flooding | | |
| Yes | 8 / 23 (34.8) | 0.88 (0.33 – 2.21) |
| No | 47 / 124 (37.9) | Ref |
| Swimming in river | | |
| Daily | 5 / 24 (20.8) | 0.38 (0.11 – 1.07) |
| Weekly | 16 / 41 (39.0) | 0.91 (0.41 – 1.95) |
| None | 34 / 82 (41.5) | Ref |
| Bathing in river | | |
| Daily | 9 / 35 (25.7) | 0.51 (0.20 – 1.21) |
| Weekly | 13 / 31 (41.9) | 1.05 (0.44 – 2.45) |
| None | 33 / 81 (40.7) | Ref |
| Washing clothes in river | | |
| Daily | 7 / 24 (29.2) | 0.70 (0.25 – 1.78) |
| Weekly | 4 / 5 (80.0) | 6.03 (0.80 – 168.44) |
| None | 44 / 118 (37.3) | Ref |
| Fishing in river | | |
| Daily | 1 / 5 (20.0) | 0.40 (0.01 – 2.98) |
| Weekly | 4 / 20 (20.0) | 0.37 (0.10 – 1.10) |
| None | 50 / 122 (41.0) | Ref |
| Other river activities | | |
| Daily | 3 / 4 (75.0) | -- |
| Weekly | 1 / 1 (100.0) | -- |
| None | 51 / 142 (35.9) | Ref |
| Animal contact* | | |
| Cats | | |
| Yes | 27 / 83 (32.5) | 0.62 (0.31 – 1.22) |
| No | 28 / 64 (43.8) | Ref |
| Dogs | | |
| Yes | 20 / 57 (35.1) | 0.85 (0.42 – 1.70) |
| No | 35 / 90 (38.9) | Ref |
| Pigs | | |
| Yes | 6 / 16 (37.5) | 1.01 (0.32 – 2.95) |
| No | 49 / 131 (37.4) | Ref |
| Poultry | | |
| Yes | 17 / 44 (38.6) | 1.08 (0.51 – 2.23) |
| No | 38 / 103 (36.9) | Ref |
| Other (sheep, squirrel) | | |
| Yes | 2 / 2 (100.0) | -- |
| No | 53 / 145 (36.6) | Ref |
| Seen a rodent (in the last 3 months) | | |
| Yes | 34 / 80 (42.5) | 1.80 (0.88 – 3.79) |
| No | 21 / 67 (31.3) | Ref |
| Handled dead rodent (in the last 3 months) | | |
| Yes | 5 / 22 (22.7) | 0.45 (0.14 – 1.24) |
| No | 50 / 125 (40.0) | Ref |
| Mode of transportation* | | |
| Vehicle | 33 / 88 (37.5) | Ref |
| Boat | 10 / 34 (29.4) | 0.70 (0.29 – 1.63) |
| Motorbike / bicycle | 15 / 33 (45.5) | 1.39 (0.61 – 3.14) |
| On foot | 9 / 37 (24.3) | 0.54 (0.22 – 1.26) |
| NA | 3 / 6 (50.0) | 1.66 (0.27 – 10.15) |
| Route system | | |
| Gravel road | 10 / 23 (43.5) | Ref |
| Logging track | 5 / 17 (29.4) | 0.56 (0.13 – 2.10) |
| Dirt road | 13 / 30 (43.3) | 0.99 (0.33 – 3.05) |
| Tarred road | 33 / 80 (41.3) | 0.91 (0.35 – 2.40) |
| Main river | 6 / 18 (33.3) | 0.66 (0.17 – 2.40) |
| Small river | 7 / 17 (41.2) | 0.91 (0.25 – 3.33) |
| NA | 1 / 4 (25.0) | 0.48 (0.02 – 4.85) |
| Average monthly income (18+) | | |
| None | 12 / 32 (37.5) | Ref |
| Below 1000 MYR | 6 / 20 (30.0) | 0.72 (0.20 – 2.39) |
| 1000 – 2000 MYR | 6 / 22 (27.3) | 0.64 (0.18 – 2.06) |
| Above 2000 MYR | 2 / 4 (50.0) | 1.64 (0.15 – 17.54) |
| Work environment (18+) | | |
| Mostly indoor | 5 / 17 (29.4) | Ref |
| Mostly outdoor | 11 / 30 (36.7) | 1.37 (0.38 – 5.40) |
| A mix of indoor / outdoor | 3 / 16 (18.8) | 0.57 (0.09 – 2.98) |
| NA | 7 / 15 (46.7) | 2.04 (0.47 – 9.53) |
| Occupational exposure (18+)* | | |
| Agricultural farming | | |
| Yes | 12 / 29 (41.4) | 1.75 (0.66 – 4.68) |
| No | 14 / 49 (28.6) | Ref |
| Fishing | | |
| Yes | 6 / 17 (35.3) | 1.13 (0.34 – 3.47) |
| No | 20 / 61 (32.8) | Ref |
| Hunting | | |
| Yes | 3 / 11 (27.3) | 0.74 (0.14 – 2.91) |
| No | 23 / 67 (34.3) | Ref |
| Livestock farming | | |
| Yes | 2 / 6 (33.3) | 1.03 (0.12 – 6.04) |
| No | 24 / 72 (33.3) | Ref |
| Logging | | |
| Yes | 3 / 9 (33.3) | 1.02 (0.19 – 4.40) |
| No | 23 / 69 (33.3) | Ref |
| Protective gear worn at work (18+)* | | |
| Any protective gear | | |
| Yes | 21 / 58 (36.2) | 1.67 (0.55 – 5.86) |
| No | 5 / 20 (25.0) | Ref |
| Closed-toe shoes | | |
| Yes | 16 / 41 (39.0) | 1.55 (0.53 – 4.82) |
| No | 10 / 37 (27.0) | Ref |
| Open-toe shoes | | |
| Yes | 9 / 24 (37.5) | 1.31 (0.46 – 3.60) |
| No | 17 / 54 (31.5) | Ref |
| Gloves (cloth, latex) | | |
| Yes | 5 / 11 (45.5) | 1.82 (0.46 – 6.90) |
| No | 21 / 67 (31.3) | Ref |
| Eye protection | | |
| Yes | 1 / 4 (25.0) | 0.71 (0.02 – 6.48) |
| No | 25 / 74 (33.8) | Ref |
| Facemask | | |
| Yes | 3 / 7 (42.9) | 1.57 (0.27 – 8.13) |
| No | 23 / 71 (32.4) | Ref |
| Other | | |
| Yes | 1 / 3 (33.3) | 1.06 (0.03 – 13.68) |
| No | 25 / 75 (33.3) | Ref |

* Primary water source, transportation mode, occupational exposure, and protective gear worn are not mutually exclusive; respondents may have selected more than one response in each category

**Supplemental Figure 1: Microagglutination testing results of 59 sera samples sent for confirmatory leptospirosis testing.** Prescreening with Leptorapide latex agglutination testing (Linnodee, Ltd., Antrim, Northern Ireland) determined 53 of these samples to be positive for leptospirosis, five inconclusive, and one negative.

**
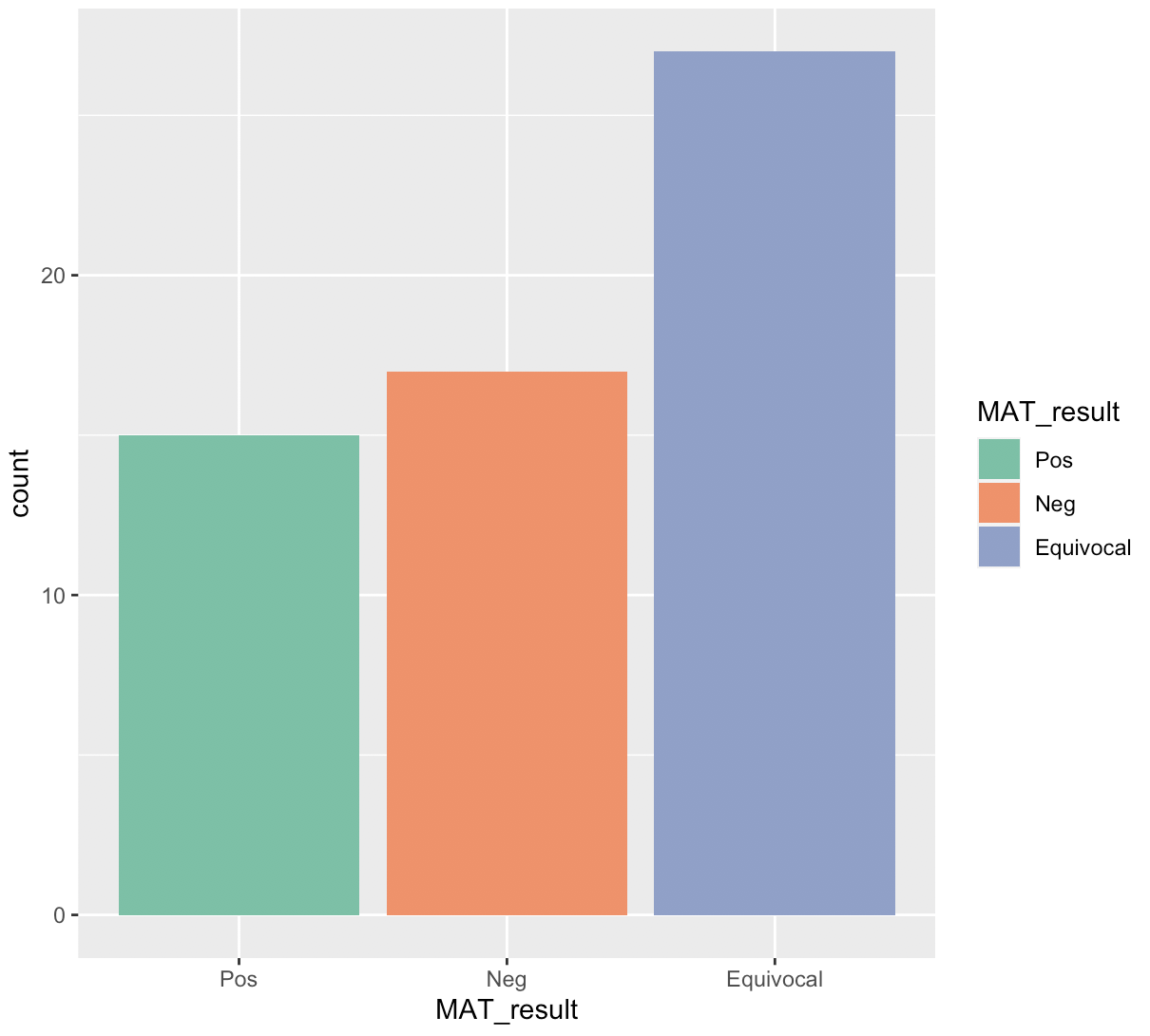

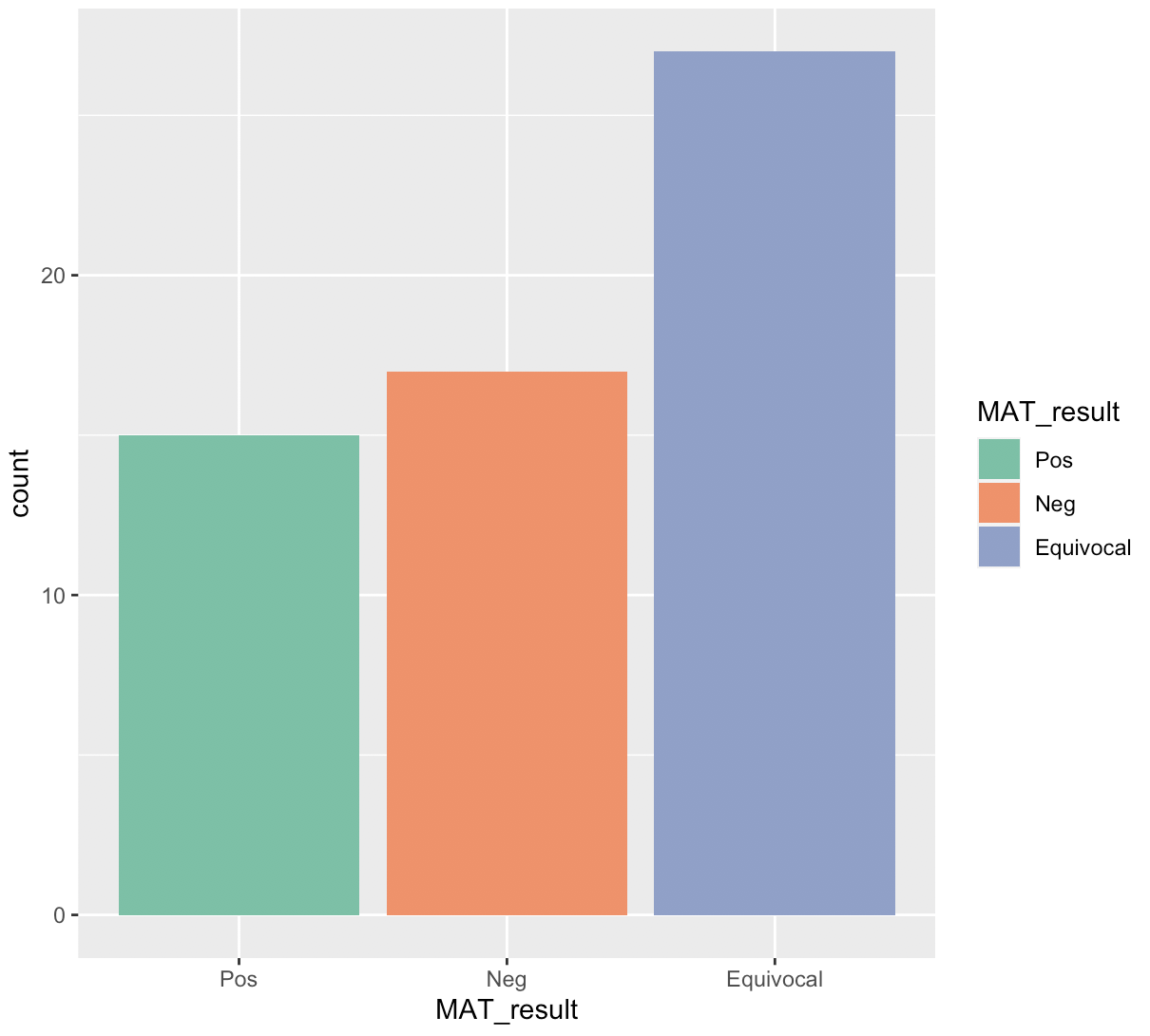
**

MAT (n = 59)

**Supplemental Figure 2: The detection fraction of different leptospirosis diagnostic tests according to days post-symptom onset for a study population across three enrolling hospitals in Sarawak, Malaysia.** Most cases (52 out of 55, 94.5%) had symptoms between day 2 to day 7. Only one case presented on day 8 and two on day 14 of febrile illness.


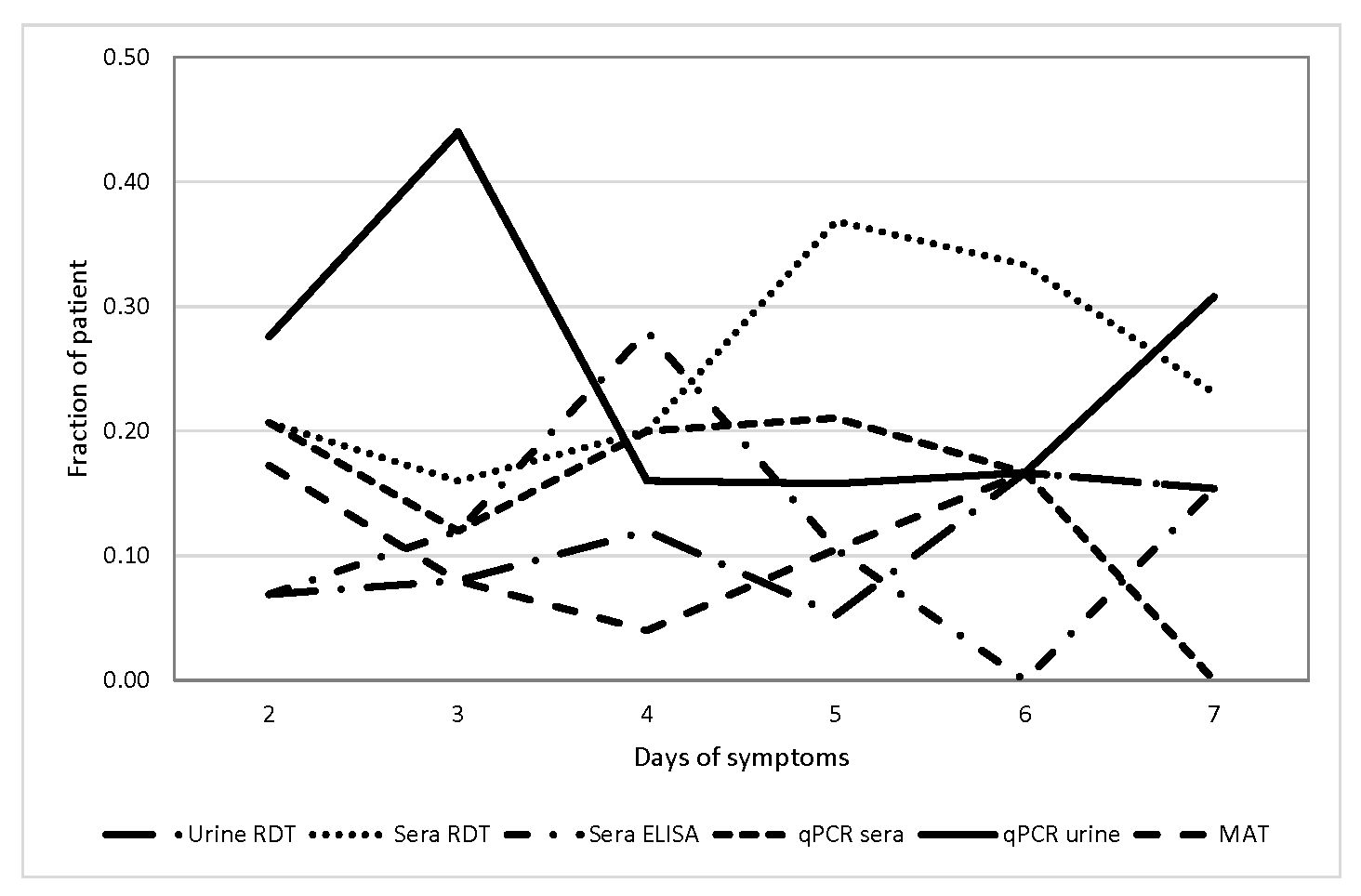


**^*^** Rapid diagnostic test Leptorapide latex agglutination test (Linnodee, Ltd., Antrim, Northern Ireland). **^†^** Microagglutination testing, as carried out by the Institute of Medical Research reference lab in Kuala Lumpur, Malaysia, only partial results available. **^‡^** *Leptospira* IgM ELISA assay (PanBio, Queensland, Australia).

**References**

1. Ahmed N, Devi SM, Valverde Mde L, Vijayachari P, Machang'u RS, Ellis WA, et al. Multilocus sequence typing method for identification and genotypic classification of pathogenic Leptospira species. Ann Clin Microbiol Antimicrob. 2006;5:28.
